# Influenza B viruses in pigs, Taiwan

**DOI:** 10.1111/irv.12588

**Published:** 2018-10-27

**Authors:** Ching‐Ping Tsai, Hsiang‐Jung Tsai

**Affiliations:** ^1^ Division of Animal Resources Animal Technology Laboratories Agricultural Technology Research Institute Hsinchu City Taiwan; ^2^ Zoonosis Research Center School of Veterinary Medicine National Taiwan University Taipei City Taiwan

**Keywords:** emerging disease, influenza virus, surveys, swine, Taiwan, Zoonosis

## Abstract

**Background:**

Influenza B viruses (IBVs) have never been isolated from natural‐infected pigs in clinical cases, although the susceptibility of domestic pigs to experimental IBV infections had been confirmed as well as IBV‐specific antibodies were detected from pigs under natural and experimental conditions.

**Objectives:**

We aimed to assess and investigate the activities for infection and circulation of IBVs in pigs.

**Methods:**

Annual active surveys for influenza have been implemented on swine populations in Taiwan since July 1998. Nasal swabs, trachea, lungs, and blood from pigs were tested using virological and serological assays for influenza. Gene sequences of influenza viral isolates were determined and characterized. Preliminary sero‐epidemiological data for influenza virus were investigated.

**Results:**

Three strains of IBV were isolated and identified from natural‐infected pigs in 2014. Genetic characterization revealed the highest identities (>99%) of molecular sequence with the contemporary IBVs belonged to the B/Brisbane/60/2008 genetic clade of Victoria lineage in the phylogenetic trees for all 8 genes. IBV‐specific antibodies were detected in 31 (0.2%; 95%CI: 0.1%‐0.2%) of 15 983 swine serum samples from 29 (2.8%; 95%CI: 1.9%‐3.9%) of 1039 farm visits under annual active surveys from 2007 through 2017. Seropositive cases have been found sparsely in 1‐5 of test prefectures every year except 2015 and 2017 as well as scattered loosely over 26 townships/districts of 11 prefectures in Taiwan cumulatively in 11 years.

**Conclusions:**

Influenza B viruse infections from humans to pigs remained sporadic and accidental currently in Taiwan but might have paved potential avenues for newly emerging zoonotic influenza in the future.

## 1 INTRODUCTION

Influenza viruses had been classified into 4 genera (A, B, C and D) in the *Orthomyxoviridae* family.^1^ Influenza A, C and newly identified D viruses (IAV, ICV and IDV) had been isolated and characterized from pigs since 1930 [A/swine/Iowa/15/1930 (H1N1)],^2^ 1981 (C/pig/Beijing/10/1981 and C/pig/Beijing/32/1981)^3^ and 2011 (D/swine/Oklahoma/1334/2011),^4^ respectively. Influenza B virus (IBV) has been thought to be primarily a human pathogen since its first identification in 1940 (B/Lee/1940) by Francis.^5^ IBVs are classified into two antigenically distinct lineages, according to the phylogenetic relationship of the hemagglutinin (HA) gene: the Victoria lineage B/Victoria/2/1987 and the Yamagata lineage B/Yamagata/16/1988.^6^, ^7^ Since 2008, most B/Victoria/2/1987 lineage viruses have belonged to the B/Brisbane/60/2008 genetic clade based on the HA gene sequences.^8^ In addition to humans as natural host and reservoir, IBVs had been sporadically isolated from pheasants (in Hungary),^9^ horses (in Japan),^10^ dogs (in 3 urban communities of Taiwan, July 1971),^11^ and seals (in the Netherlands, B/seal/Netherlands/1/1999)^12^ as well. To our knowledge, IBVs have never been isolated from natural‐infected pigs in clinical cases. Experimental infection of 12 piglets with infectious IBV (B/Budapest/10/1965) on February 1, 1968, was first conducted in Hungary.^13^ The susceptibility of the domestic pig to IBV was confirmed. A serological survey for Midwest domestic swine herds of the United States from 2010 to 2012 was conducted. Meanwhile, an experimental challenge study was performed in pigs using two representative IBVs: B/Yamagata/16/1988 and B/Brisbane/60/2008.^14^ The susceptibility of domestic pigs to IBV infection in the US study supports and extends former observations made in the 1960s.^13^

In this report, we presented gene sequence analyses and comparisons for IBVs isolated from natural infections in Taiwanese swine herds under active survey of influenza in 2014. Preliminary epidemiological investigations of antibodies against swine strain of IBV (sIBV) in our collection of sera under annual active surveys during 2007‐17 were discussed.

## 2 MATERIALS AND METHODS

### 2.1 Sample studied

Official veterinarians at local animal disease control centers had involved and visited swine farms according to predetermined schedule and sampling strategy. Nasal swabs, trachea, lungs, and blood were collected from pigs. Case history of each visited farm was collected via questionnaire. Until the end of 2017, specimens of around 30 000 pigs from more than 1500 farm visits were submitted with case history to our laboratory for influenza virus isolation and antibody detection. The 15 983 swine serum samples were collected from 1039 farm visits under active surveys during 2007‐17.

### 2.2 Virus isolation and identification

Viral specimens were processed and inoculated into Madin‐Darby canine kidney (MDCK) cells (BCRC 60004 obtained from ATCC CCL‐34, Food Industry Research and Development Institute, Hsinchu, Taiwan) and 10‐day‐old chicken embryonated eggs (Animal Drugs Inspection Branch of Animal Health Research Institute or JD‐SPF Biotech, Miaoli, Taiwan), respectively.^15^ After predetermined incubation period, culture supernatant and allantoic fluid were mixed with 0.5% (v/v) rooster or male turkey erythrocyte suspension to prescreen any probable hemagglutinating agents. Mouse anti‐influenza A (Cat. No. MAB8251) and B (Cat. No. MAB8661) virus nuclear protein monoclonal antibodies (EMD Millipore Corporation, Temecula, CA) were applied to cold 80% acetone‐fixed cell culture for type determination by immunofluorescence (IF) staining. Ribonucleic acid (RNA) was extracted from viral isolates using the QIAamp Viral RNA Mini Kit (QIAGEN, Hilden, Germany). Several sets of primer specific for detection and amplification of IAV genes and IBV genes (primer names and sequences provided in: Table S1) were used in reverse transcription‐polymerase chain reaction (RT‐PCR).

### 2.3 Sequence analyses

The resultant RT‐PCR products were purified using a QIAquick PCR purification kit (QIAGEN, Hilden, Germany) and sequenced with IBV gene‐specific primers (primer names and sequences provided in: Table S1). Nucleotide sequences were determined with an ABI PRISM BigDye Terminator Cycle Sequencing Ready Reaction Kit (V3.1) in an ABI 3730 DNA analyzer (Applied Biosystems, Foster City, CA). Fragments and contigs of gene sequences were processed, built and assembled using EditSeq [version 9.1.0 (109) 418], SeqBuilder [version 9.1.0 (109)] and SeqMan [version 9.1.1 (4) 418] programs, respectively, in the LaserGene biocomputing software package (DNASTAR, Madison, WI). Using the Basic Local Alignment Search Tool (BLASTN 2.2.19), we retrieved corresponding sequences with the highest identities from publicly available sequence database (GenBank and EpiFlu) in the website of National Center for Biotechnology Information (NCBI) ( https://www.ncbi.nlm.nih.gov/) and Global Initiative on Sharing All Influenza Data (GISAID) ( https://www.gisaid.org/).

### 2.4 Phylogenetic analyses

Corresponding gene sequences of B/Lee/1940, B/Victoria/02/1987, B/Yamagata/16/1988, 4 World Health Organization (WHO) recommended vaccines implemented in Taiwan from 2009 through 2016 [B/Brisbane/60/2008 (2009‐12), B/Wisconsin/01/2010 (2012‐13), B/Massachusetts/02/2012 (2013‐15) and B/Phuket/3073/2013 (2015‐16)],^16^ the BLAST‐searched best‐matching viruses as well as Victoria‐like and Yamagata‐like IBVs isolated from 2009 through 2016 in Taiwan were retrieved from GenBank and EpiFlu. Sequence alignments were executed using the ClustalW program embedded in Molecular Evolutionary Genetics Analysis software (MEGA, version 7.0; http://www.megasoftware.net/).^17^ Phylogenetic trees were constructed using the maximum‐likelihood method. Best nucleotide substitution models were used: the Hasegawa‐Kishino‐Yano model with a gamma distribution (HKY + G) for HA gene; and the Tamura 3‐parameter model with gamma distribution (T92 + G) for neuraminidase (NA) and 6 internal protein genes, respectively. Reliability of the tree topology was assessed by bootstrap analysis with 1000 replications.^18^

### 2.5 Serological analyses

Hemagglutination‐inhibition (HI) assays^15^ were used to detect antibodies against IBVs in swine serum samples. To eliminate non‐specific inhibitors, swine sera were treated with 100 U/mL of receptor‐destroying enzyme (Cholera filtrate, Cat. No. C8772; Sigma‐Aldrich/Merck KGaA, Darmstadt, Germany) at 37°C overnight, heat‐inactivated at 56°C for 30 minutes, and absorbed with 10% (v/v) rooster or male turkey erythrocytes at 4°C for 1 hour. A serial 1:2 dilution of treated serum samples was tested using 4 hemagglutinating units of sIBVs (B/swine/Pingtung/58‐15/2014, B/swine/Tainan/77‐5/2014 and B/swine/Taoyuan/78‐1/2014, respectively) and 0.5% (v/v) rooster or male turkey erythrocytes. The reciprocal of the highest serum dilution that completely inhibits hemagglutination is considered HI titer. The virus neutralization (VN) assays^15^ were performed to confirm the results of the HI assays. Untreated original aliquots of HI‐positive swine sera were 10‐fold‐diluted with sterilized phosphate‐buffered saline (pH 7.2 ± 0.2) and then heat‐inactivated at 56°C for 30 minutes. A serial 1:2 dilution of inactivated serum samples was assayed using above‐mentioned reference viruses (100 TCID_50_/50 μL) on MDCK cell line cultures.

## 3 RESULTS

### 3.1 Isolation and identification of influenza B virus from pigs

The nasal swabs of fattening pigs collected from 3 swine farms in 2014 had been confirmed to harbor IBVs. Case history for swine herds where 3 sIBVs isolated is provided in Table S2. Culture supernatant of MDCK cell line and allantoic fluid from embryonated eggs were collected after incubation of viral specimens, respectively. Hemagglutinating agents were detected with erythrocyte suspension. Fluorescence signals could be displayed on fixed MDCK cells stained with antibodies against nucleoprotein (NP) of IBV only but not IAV. Expected RT‐PCR products were amplified by primers specific for IBV genes only. Gene sequences of 3 sIBVs are very highly similar but not fully identical to each other. Several variations exist in coding region of HA, NA, matrix protein 1 (M1), polymerase acidic protein (PA) and polymerase basic protein 1 (PB1) genes, respectively. BLAST results (data provided in: Table S3) indicated that 3 sIBVs have the highest identities (>99%) of nucleotide sequence for HA and NA genes with one human IBV (B/Taiwan/113/2014), which collected on April 10, 2014,^16^ as well as for other 6 internal protein genes with the contemporary Victoria‐like strains.

### 3.2 Phylogenetic relationship between swine and human strains of influenza B virus

**Figure 1:**
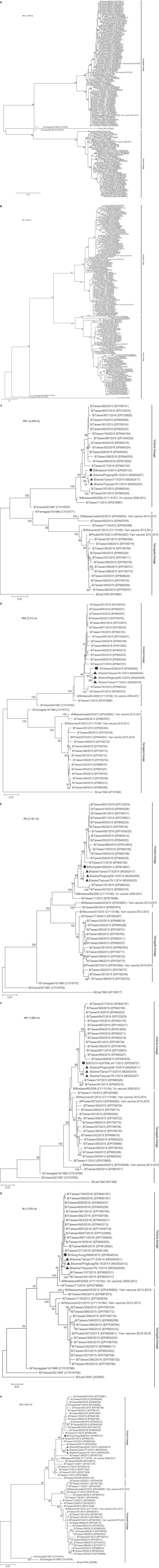
Molecular phylogenetic analyses of corresponding genes between swine and human strains of influenza B virus. Evolutionary analyses were conducted in MEGA7.^17^ In addition to corresponding genes of B/Lee/1940, B/Victoria/02/1987, B/Yamagata/16/1988, 4 vaccine strains recommended by WHO and implemented in Taiwan from 2009 through 2016 (Yam denotes Yamagata and Vic denotes Victoria),^16^ 3 sIBVs (denoted as ▲) and one of the BLAST‐searched best‐matching viruses (denoted as ●), 122 HA genes (panel A), 151 NA genes (panel B), 31 PB1 genes (panel C), 31 PB2 genes (panel D) and 32 PA genes (panel E), 32 NP genes (panel F), 32 M genes (panel G) and 49 NS genes (panel H) of human IBVs isolated from 2009 through 2016 in Taiwan were involved, respectively. The numbers of nucleotide analyzed in corresponding genes are indicated on panels. The evolutionary history was inferred using the maximum‐likelihood method based on the Hasegawa‐Kishino‐Yano model with a gamma distribution (HKY + G) for HA genes and the Tamura 3‐parameter model with gamma distribution (T92 + G) for NA and 6 internal protein genes, respectively. Reliability of the tree topology was assessed by bootstrap analysis with 1000 replications.^18^ Only branches with bootstrap values >75% are indicated on phylogenetic trees. Scale bar indicates nucleotide substitutions per site. Accession numbers are listed in parentheses

Three sIBVs are grouped with B/Taiwan/13/2013 and B/Taiwan/113/2014 in the same cluster of the B/Brisbane/60/2008 genetic clade of Victoria lineage from the HA tree of 133 IBVs and the NA tree of 162 IBVs (Figure 1, panels A,B), respectively. In the phylogenetic trees of other 6 internal protein genes (Figure 1, panels C‐H), 3 sIBVs and the BLAST‐searched best‐matching viruses for corresponding genes are distributed into the subtree of B/Brisbane/60/2008‐like viruses, respectively.

### 3.3 Amino acid sequence analysis of swine influenza B virus

Comparisons with B/Taiwan/113/2014 (the best‐matching virus for HA and NA genes) revealed that all sIBVs have N197D and S520T substitutions but B/swine/Tainan/77‐5/2014 and B/swine/Taoyuan/78‐1/2014 carry additional I424M substitution in HA protein as well as B/swine/Tainan/77‐5/2014 harbor F172L substitution in NA protein. When compared with B/Brisbane/60/2008, sequence variations in HA, NA, NP, M1, non‐structural protein 1 (NS1), PA, PB1 and polymerase basic protein 2 (PB2) proteins have been identified in all sIBVs or some of them. In HA protein, 4 amino acid substitutions (V124A, I146V, N197D, and D525E) were observed in all sIBVs but I424M substitution only in B/swine/Tainan/77‐5/2014 and B/swine/Taoyuan/78‐1/2014 (data provided in: Table S4). In addition to F172L substitution only in B/swine/Tainan/77‐5/2014, 4 amino acid substitutions (S295R, N340D, E358K, and M369I) exist in NA protein of all sIBVs (data provided in: Table S4). Besides, all sIBVs have 1 substitution (I9M) in NP, 1 substitution (V474I) in PB1 protein, 3 substitutions (K107N, V190A, and S205L) in NS1 protein, and 3 substitutions (R115K, I492T, and S630N) in PB2 protein, respectively. M86V substitution in M1 protein was observed only in B/swine/Pingtung/58‐15/2014. As to PA protein, all sIBVs comprise N714S substitution but B/swine/Pingtung/58‐15/2014 possesses additional I428M substitution.

### 3.4 Serological data of influenza B virus in swine populations

Based on a conservative HI cut‐off titer of 1:40 for seropositivity,^14^, ^19^ antibodies against sIBVs were detected in 31 (0.2%; 95% CI: 0.1%‐0.2%) of 15 983 swine serum samples from 29 (2.8%; 95% CI: 1.9%‐3.9%) of 1039 farm visits collected from 2007 through 2017. Positive samples have no more than 160 HI titers, and 18 (58.1%) of them showed antibody titers ≥80. All HI‐positive samples were further confirmed by the VN assays. In 11 years, seropositive cases have been found sparsely in 1‐5 of test prefectures every year except 2015 and 2017 as well as scattered loosely over 26 townships/districts of 11 prefectures in Taiwan cumulatively (Table 1). Notably, the first one of sIBVs was isolated from swine farm in the Wandan Township of Pingtung County (data provided in: Table S2), but none of positive swine serum samples was found from specimens collected at the same time and any subsequent visits thereafter. As for the other 2 swine farms which IBVs were isolated, none of IBV‐specific antibodies had ever detected from serum samples collected at the same farms and any other farms in the same township or district.

**Table 1:** Seropositive^a^ cases on swine farms for swine strains of influenza B virus at prefecture level in Taiwan

Year	Swine sera		Swine farm		Prefecture	
	+/Test	+%	+/Test	+%	+/Test	+%
2007	1/992	0.1	1/76	1.3	1/25 (Kinmen)	4.0
2008	2/1290	0.2	2/69	2.9	2/14 (Pingtung, Tainan)	14.3
2009	6/1695	0.4	5/108	4.6	4/18 [Yilan, Yunlin (2 townships), Chiayi, Pingtung]	22.2
2010	6/2062	0.3	6/139	4.3	5/25 [Yunlin, Yilan (2 townships), Changhua, Kaohsiung, Tainan]	20.0
2011	3/1490	0.2	3/95	3.2	3/16 (Yunlin, Chiayi, Tainan)	18.8
2012	5/1388	0.4	4/91	4.4	4/16 (Pingtung, Chiayi, Yilan, Kaohsiung)	25.0
2013	2/1359	0.1	2/88	2.3	2/16 (Yunlin, Changhua)	12.5
2014	2/1309	0.2	2/87	2.3	2/18 (Tainan, Taitung)	11.1
2015	0/1466	0	0/94	0	0/18	0
2016	4/1460	0.3	4/96	4.2	4/22 (Matsu, Tainan, Hualien, Yunlin)	18.2
2017	0/1472	0	0/96	0	0/20	0
Sum	31/15 983	0.2	29/1039	2.8	11/25	44.0

a A conservative HI cut‐off titer of 1:40 considered as positive for swine serum sample.^14^, ^19^ Swine farm recorded as positive when at least one of swine serum samples have 40 or more HI titers. All HI‐positive samples were further confirmed by the VN assays.

## 4 DISCUSSION

Even IBV is thought to be primarily a human pathogen, several serological, molecular, and experimental infection evidences have supported that pigs may serve as one of natural reservoirs. Ran et al^14^ presented quite high seropositivity of IBV‐specific antibodies in 38.5% (20/52) of sampled farms, and 7.3% (41/560) of tested swine serum samples on US Midwest domestic swine farms from 2010 to 2012 in contrast to our findings [31 (0.2%) of 15 983 serum samples from 29 (2.8%) of 1039 farm visits] on Taiwanese swine populations from 2007 through 2017. Experimental infections^14^ indicated that B/Victoria lineage virus replicated and transmitted more efficiently than B/Yamagata lineage virus in pigs. Although the partial sequences of the NS gene were detected in 3 nasal swab samples of pigs, the efforts to isolate IBVs are fail.^14^ In this study, however, IBVs were isolated from pigs in 3 commercial swine farms of Taiwan under active survey in 2014. Furthermore, there might be a mere coincidence that 3 sIBVs are all B/Brisbane/60/2008‐like viruses of Victoria lineage. It implies the finding on clinical cases in Taiwan seems to support the experimental infection observations on difference between both lineages of IBVs.^14^ Therefore, transmissibility and pathogenesis of our sIBVs on pigs await further experimental and clinical investigations.

Case history for swine herds where 3 sIBVs were isolated manifested that asymptomatic influenza infections occurred independently in pigs on swine farms, which are small scale as well as have poor animal health management, biosecurity, and hygiene practices, in different parts of Taiwan (data provided in: Table S2). Our long‐term experiences indicated that most of IAVs were isolated from symptomless or mild sick pigs under annual active survey. Therefore, it would not be surprised that IBVs could be isolated from subclinical pigs in this study. Besides, a matter worthy of note is that none of personnel in those 3 commercial swine farms had received seasonal influenza vaccine. In Taiwan, poultry farmers and animal health inspectors had been included in target groups of government‐funded seasonal influenza vaccination campaign launched by Taiwan Center for Disease and Control (TCDC). However, questionnaire collected from our annual active surveys indicated that personnel in only 13.95% (=12/86), 12.79% (=11/86), 29.17% (=28/96) and 22.68% (=22/97) of swine farms have injected with seasonal influenza vaccine in 2014, 2015, 2016, and 2017, respectively. The persons who will close contact with pigs and have never received influenza vaccines, therefore, might be intermediate vehicles for disseminating IBVs to pigs.

In Taiwanese human populations, influenza B predominated over influenza A in several influenza seasons and some of the interseason periods as well as two lineages changed rotationally or cocirculated in different periods.^20^, ^21^ Hsieh et al^22^ reported the waves and high weekly percentage of IBVs appeared in 6 periods: 2008/31‐42, 2010/08‐38, 2011/04‐15, 2011/29‐34, 2011/39‐2012/13, and 2014/03‐18 (y/wk). Kuo et al^16^ presented lineage‐specific statistics for IBVs that circulated in Taiwan during 2003‐2014. More than 50% of Victoria‐like isolations dominated in 2005/2006, 2006/2007, 2009/2010, 2012/2013, and 2013/0214 influenza seasons, respectively. As for B/Taiwan/113/2014, one of the best‐matching viruses of Victoria lineage for sIBVs, according to BLAST results (data provided in: Table S3), was collected on April 10, 2014 (i.e, 2014/15 y/wk). Three sIBVs were isolated on July 4, October 24, and October 27, 2014, respectively. To consider them together, Victoria‐like IBVs circulated in weeks 3‐18 of 2013/0214 influenza season, therefore, might be sources for transmission from humans to pigs in Taiwan.

Phylogenetic analyses revealed also that our sIBVs might be originated from B/Taiwan/113/2014 or the contemporary human IBVs in the same cluster of the B/Brisbane/60/2008 genetic clade (as seen as the Victoria Clade III in the study of Kuo et al)^16^ in Victoria lineage (Figure 1, panels A‐H). Compared with B/Brisbane/60/2008, amino acid sequence variations relevant to antigenicity, evolutionary signatures, and biological characteristics were examined. In IBV HA protein, 4 antigenic sites were identified: 120‐loop (positions 116‐137), 150‐loop (positions 141‐150), 160‐loop (positions 162‐167), and 190‐helix (positions 194‐202), respectively.^23^ Diversity in the 120‐loop was frequently found in recent IBVs during 2010‐15 in Italy, Malaysia, China, and Thailand.^24^ All sIBVs carry V124A substitution in the 120‐loop, which has been also found in one of Italian IBVs (B/Milano/35CA/2016).^24^ I146V substitution in the 150‐loop of all sIBVs has been also observed in 89.6% of B/Victoria clade 1 strains isolated in Italy during the period 2010‐15,^25^ several influenza B‐Victoria lineage viruses isolated in Cambodia in 2010‐11^26^ as well as 67 Victoria‐1 viruses isolated in Malaysia, 2012‐14.^27^ None of Amino acid substitutions in the 160‐loop were found in all sIBVs.

The 190‐helix comprising amino acid residue 194‐202 contains a glycosylation site.^23^ The N‐linked glycosylation plays a major role in stabilizing the HA structure, to protect the HA protein from being hydrolyzed by the enzyme and to evade antibody recognition. Residue 197 in the 190‐helix was changed from N to D in all sIBVs. This N197D mutation in N‐glycosylation motif (N‐X‐S/T) is associated with a loss of glycosylation site in HA1 and has been found in at least one of Taiwanese Victoria‐like human IBVs (B/Taiwan/2894/2006) in 2006/2007 season compared with B/Malaysia/2506/2004 vaccine,^16^ as well as 3 Zhejiang isolates of 2010 (B/Zhejiang/41/2010, B/Zhejiang/49/2010 and B/Zhejiang/429/2010) in China^28^ and 3 Italian Victoria‐like human IBVs (B/Milano/40NIC/2016, B/Milano/28CA/2016 and B/Milano/35CA/2016)^24^ compared with B/Brisbane/60/2008 vaccine. Several reports have demonstrated that egg‐adaptive mutations might lead to lost glycosylation.^29^, ^30^ By contrast, loss of this N‐glycosylation site can also occur in strains cultured only in MDCK cells and some egg‐cultured strains can retain it.^30^ Even if egg‐adaptive mutations could not be ruled out, N197D mutation in our sIBVs might occur in the antigenic drift descendants of possible viral sources (B/Taiwan/113/2014‐like virus) before accidental infection of the pigs or the result of adaptation during infection in pigs.

In NA protein, drug‐resistant amino acids have been reported, such as the oseltamivir‐resistant mutations E105K, G109E, E119A, D197E, D198N, I221V, I222T, H274Y, R292K, and N294S; zanamivir‐resistant mutations G109E, E119A, R152K, D197E, and I222T; and peramivir‐resistant mutations E105K, D197E, and H273Y(H274Y).^16^ None of them was found on all three sIBVs. It suggests that our sIBVs are sensitive to NA inhibitors. However, 3 of major signature amino acid substitutions (S295R, N340D, and E358K), which shared by V1A‐2 subclade viruses of the 2012‐14 Malaysian IBVs, based on the recent WHO genetic groupings^27^ were observed from all sIBVs (data provided in: Table S4). As for biological importance of other amino acid alternations found in proteins of all sIBVs, especially cross‐species transmission and adaptation, further clarifications and investigations are required.

Implementing avian influenza vaccination for poultry was prohibited in Taiwan^31^ because of considerations on control measures and prevention policy of animal influenza for livestock industries issued from COA. The prohibition had applied to swine populations as well. Antibodies against any kind of influenza viruses detected from pigs should be real reflexes and responses of natural influenza infection. The interpretation of serological data on active influenza outbreaks would not be interfered and misled by vaccine‐induced antibodies. In this study, seropositive cases for sIBVs scattered loosely and cumulatively over 11 (44.0%; 95% CI: 26.5%‐63.0%) of 25 prefectures in 11 years (Table 1). IBV infections in pigs had occurred in no more than 5 prefectures or 25.0% (95% CI: 10.3%‐49.8%) of test prefectures every year except 2015 and 2017. Meanwhile, no more than 6 positive farms found in positive prefectures. In positive townships/districts, no more than 2 farms are seropositive. In all positive farms, most of them have only one positive swine serum and no more than 2 in the remaining farms. In the period of study, therefore, positive cases for isolation of IBVs and detection of IBV‐specific antibodies were not confined to localized area and found infrequently. The trend for temporal increment and spatial expansion of seropositive cases year by year was not observed. The findings mean that transmission of IBVs remained probably sporadic and accidental at the human‐swine interface as none of available evidences that swine workers were positive for IBV. Public health threats, however, might be considered because swine populations have served as a reservoir of IAV genes that have circulated in humans.^32^ Human IBVs would be kept in swine populations and provided as genetic sources for reassortment of novel influenza viral variants. Even only sporadic cases of IBV were found during our study period in Taiwanese swine populations, reassortment events of swine IBV with other kind of influenza viruses (IAV and any unpredictable types) in pigs might be happened to create newly emerging zoonotic influenza in the future.

In conclusion, public health concerns might arise from the finding that IBVs could be transmitted naturally from humans to pigs, even cross‐species infections are sporadic and accidental currently in Taiwan. It implies a cycle of human‐swine‐human transmission could be or might have been initiated as some of IAVs did in human and swine populations.^33^ Therefore, IBV infections in Taiwanese pigs should deserve much more attention, better surveillance, and further detailed investigations.

## CONFLICT OF INTEREST

The authors of this article report no conflict of interests.

## Supporting information


** **
