# Supplementary material for: Influenza B viruses in pigs, Taiwan
Source: Influenza Other Respir Viruses. 2018 Oct 27;13(1):91–105. doi: 10.1111/irv.12588 (PMC6304316; doi:10.1111/irv.12588)
Supplement: Supplementary file 1 [file IRV-13-91-s001.docx]

Supporting Information

Table S1. Primers used in this study

| Primer name | Primer sequence (5′ to 3′) | Purpose | Source |
| --- | --- | --- | --- |
| M30F | TTCTAACCGAGGTCGAAACG | Detection of influenza A viral M gene | Poon LL et al.^1^ |
| M264R2 | ACAAAGCGTCTACGCTGCAG |  |  |
| H1F | gggACATgTTACCCAggAgAT | Detection and subtyping of influenza A viral H1 HA gene | Choi YK et al.^2^ |
| H1R | gCATTgTATgTCCAAATATCCA |  |  |
| N1F | ggTTCCAAAggAgACATTTTTg | Detection and subtyping of influenza A viral N1 NA gene |  |
| N1R | CTATCCAAACACCATTgCCATA |  |  |
| H1R (411) | CTgCTTgACCTCTCACTTTgg | Detection and subtyping of influenza A viral H1 HA gene | Choi YK et al.^3^ |
| H3-175f | CARATTGARGTGACHAATGC | Detection and subtyping of influenza A viral H3 HA gene | Lee MS et al.^4^ |
| H3-896r | GGTGCATCTGAYCTCATTA |  |  |
| AN2B | ggTgACgAgAgAACCTTATg | Detection and subtyping of influenza A viral N2 NA gene | Takao S et al.^5^ |
|  |  |  |  |
| AN2CII | CCTgAgCACACATAACTggA |  |  |
| B/HA98 | ATAACATCGTCAAACTCACC | Detection of influenza B viral HA gene | Tsai HP et al.^6^ |
| B/HA836 | GCACCATGTAATCAACAACA |  |  |
| B/NA1 | GCTACCTTCAACTATACAAACG | Detection of influenza B viral NA gene |  |
| B/NA2 | AACGAGGGTATGTCCACTCC |  |  |
| B/PB2-F | gcaggaataccaagagaatc | Detection of influenza B viral PB2 gene | Lee EY et al.^7^ |
| B/PB2-R | tcttgagaaaataccatgca |  |  |
| B/PB1-F | tagtagttgaaaacttccc | Detection of influenza B viral PB1 gene |  |
| B/PB1-R | cagtaacttttctttttgctc |  |  |
| B/PB1AF | TATTCGTCTCAGGGAGCAGAAGCGGAGCCTTTAAGATG | Full-length amplification of influenza B viral genes | Hoffmann E et al.^8^ |
| B/PB1AR | TATTCGTCTCGATGCCGTTCCTTCTTCATTGAAGAATGG |  |  |
| B/PB1BF | TATTCGTCTCGGCATCTTTGTCGCCTGGGATGATGATG |  |  |
| B/PB1BR | ATATCGTCTCGTATTAGTAGAAACACGAGCCTT |  |  |
| B/PB2AF | TATTCGTCTCAGGGAGCAGAAGCGGAGCGTTTTCAAGATG |  |  |
| B/PB2AR | TATTCGTCTCTCTCATTTTGCTCTTTTTTAATATTCCCC |  |  |
| B/PB2BF | TATTCGTCTCATGAGAATGGAAAAACTACTAATAAATTCAGC |  |  |
| B/PB2BR | ATATCGTCTCGTATTAGTAGAAACACGAGCATT |  |  |
| B/PA3AF | TATTCGTCTCAGGGAGCAGAAGCGGTGCGTTTGA |  |  |
| B/PA3AR | TATTCGTCTCCCAGGGCCCTTTTACTTGTCAGAGTGC |  |  |
| B/PA3BF | TATTCGTCTCTCCTGGATCTACCAGAAATAGGGCCAGAC |  |  |
| B/PA3BR | ATATCGTCTCGTATTAGTAGAAACACGTGCATT |  |  |
| B/HAF | TATTCGTCTCAGGGAGCAGAAGCAGAGCATTTTCTAATATC |  |  |
| B/HAR | ATATCGCTCCGTATTAGTAGTAACAAGAGCATTTTTC |  |  |
| B/NPF | TATTCGTCTCAGGGAGCAGAAGCACAGCATTTTCTTGTG |  |  |
| B/NPR | ATATCGTCTCGTATTAGTAGAAACAACAGCATTTTTTAC |  |  |
| B/NAF | TATTCGTCTCAGGGAGCAGAAGCAGAGCA |  |  |
| B/NAR | ATATCGCTCCGTATTAGTAGTAACAAGAGCATTTT |  |  |
| B/MF | TATTCGTCTCAGGGAGCAGAAGCACGCACTTTCTTAAAATG |  |  |
| B/MR | ATATCGTCTCGTATTAGTAGAAACAACGCACTTTTTCCAG |  |  |
| B/NSF | TATTCGTCTCAGGGAGCAGAAGCAGAGGATTTGTTTAGTC |  |  |
| B/NSR | ATATCGTCTCGTATTAGTAGTAACAAGAGGATTTTTAT |  |  |
| B/HA30F | CTACTCATGGTAGTAACATCC | Sequencing of influenza B viral HA gene | Jumat MR et al.^9^ |
| B/HA749R | YGGGAAGCCACCAATCTGAGAAAC |  |  |
| B/HA471F | ACCTCAGGATCTTGCCCTAACG |  |  |
| B/HA1169R | TGTGTATCCGTGCCAACCTGCAAT |  |  |
| B/HA999F | AAAGCCATAGGAAATTGCCCA |  |  |
| B/HA1840R | TCAATAACGTTTCTTTGTAAT |  |  |
| B/NA21F | GCTACCTTCAACTATACAAACG | Sequencing of influenza B viral NA gene |  |
| B/NA568R | TACCATCATGGCATGCGGA |  |  |
| B/NA361F | GCTCCCTTGATAATAAGGGAACC |  |  |
| B/NA838R | ATGTTCTACTCTTCCTGTTGG |  |  |
| B/NA716F | GGGGGARATTGTTATCTTATG |  |  |
| B/NA1505R | TTTCAGAAACAATTAAKTTCAGTAAGG |  |  |
| B/NS376R | CTGGTGTTGAAGGGTAAT | Sequencing of influenza B viral NS gene |  |
| B/NS700R | ATCTTCTTCATCCTCCACTGTAA |  |  |
| BNPF5 | AGCAGAAGTACAGCATTTTCTTG | Sequencing of influenza B viral NP gene | TewawongN et al.^10^ |
| BNPR916 | TCTTCAATGTCTGCAATCCCTGG |  |  |
| BNPF786 | GGCAGACAGAGGGCTATTGAGAG |  |  |
| BNPR3 | AGTAGAAACAACAGCATTTTTTA |  |  |
| BM250f | GAGACACAATTGCCTACCTGCTT | Sequencing of influenza B viral M gene | Byarugaba DK et al.^11^ |
| BM250r | TTCTTTCCCACCGAACCAAC |  |  |

Table S2. Case history for swine herds where 3 swine strains of influenza B virus isolated

| Case no. | A14-58-15 | A14-77-5 | A14-78-1 |
| --- | --- | --- | --- |
| Visit/Collection date | July 4, 2014 | October 27, 2014 | October 24, 2014 |
| Farm location | Wandan Township of Pingtung County (South part of Taiwan mainland) | Houbi District of Tainan City (South part of Taiwan mainland) | Yangmei Township of Taoyuan County (North part of Taiwan mainland) |
| Herd pattern | Farrow-to-finish | Farrow-to-finish | Fattening |
| Number of pigs raised on farm | 1,800 (200 breeders and 1,600 fatteners) | 300 (30 breeders, 170 fatteners and 100 nursery pigs) | 479 fatteners |
| Respiratory symptom in pigs | Not observed | Not observed | Not observed |
| Influenza-like illness in personnel | None presented | None presented | None presented |
| Biosafety measures | Entrance control and disinfection while pass in and out of farm | Entrance control of farm only | Entrance control and disinfection while pass in and out of farm and piggery |
| Influenza vaccination of personnel | None received | None received | None received |
| Specimens collected | Nasal swabs and blood of 15 fatteners | Nasal swabs and blood of 15 fatteners | Nasal swabs and blood of 15 fatteners |

Table S3. The best-matching viruses at individual corresponding genes for 3 swine strains of influenza B virus

| Gene* | Length | Virus with the highest identity^†^ | % Identity |
| --- | --- | --- | --- |
| Virus name: B/swine/Pingtung/58-15/2014 | | | |
| PB1 | 2,259nt [MG002407]^‡^ | B/Brisbane/74/2014 [EPI630124] | 99.7 |
| PB2 | 2,313nt [MG002408] | B/Vietnam/5258/2015 [EPI825536] | 99.7 |
| PA | 2,181nt [MG002409] | B/Rochester/0093/2013 [EPI812691] | 99.8 |
| HA | 1,758nt [MG002410] | B/Taiwan/113/2014 [EPI534678] | 99.8 |
| NP | 1,683nt [MG002411] | B/South Australia/11/2012 [EPI539727] | 99.7 |
| NA | 1,408nt [MG002412] | B/Taiwan/113/2014 [EPI534677] | 99.9 |
| M | 1,076nt [MG002413] | B/Hong Kong/3608/2012 [EPI904914] | 99.7 |
| NS | 1,027nt [MG002414] | B/Hong Kong/3608/2012 [EPI905147] | 99.7 |
| Virus name: B/swine/Tainan/77-5/2014 | | | |
| PB1 | 2,259nt [MG002415] | B/Brisbane/74/2014 [EPI630124] | 99.7 |
| PB2 | 2,313nt [MG002416] | B/Vietnam/5258/2015 [EPI825536] | 99.7 |
| PA | 2,181nt [MG002417] | B/Rochester/0093/2013 [EPI812691] | 99.8 |
| HA | 1,758nt [MG002418] | B/Taiwan/113/2014 [EPI534678] | 99.7 |
| NP | 1,683nt [MG002419] | B/South Australia/11/2012 [EPI539727] | 99.7 |
| NA | 1,408nt [MG002420] | B/Taiwan/113/2014 [EPI534677] | 99.8 |
| M | 1,076nt [MG002421] | B/Hong Kong/3608/2012 [EPI904914] | 99.8 |
| NS | 1,027nt [MG002422] | B/Hong Kong/3608/2012 [EPI905147] | 99.7 |
| Virus name: B/swine/Taoyuan/78-1/2014 | | | |
| PB1 | 2,259nt [MG002423] | B/Brisbane/74/2014 [EPI630124] | 99.7 |
| PB2 | 2,313nt [MG002424] | B/Vietnam/5258/2015 [EPI825536] | 99.7 |
| PA | 2,181nt [MG002425] | B/Rochester/0093/2013 [EPI812691] | 99.8 |
| HA | 1,758nt [MG002426] | B/Taiwan/113/2014 [EPI534678] | 99.7 |
| NP | 1,683nt [MG002427] | B/South Australia/11/2012 [EPI539727] | 99.7 |
| NA | 1,408nt [MG002428] | B/Taiwan/113/2014 [EPI534677] | 99.9 |
| M | 1,076nt [MG002429] | B/Hong Kong/3608/2012 [EPI904914] | 99.8 |
| NS | 1,027nt [MG002430] | B/Hong Kong/3608/2012 [EPI905147] | 99.7 |

*PB1: polymerase basic protein 1, PB2: polymerase basic protein 2, PA: polymerase acidic protein, HA: hemagglutinin, NP: nucleoprotein, NA: neuraminidase, M: matrix protein, NS: non-structural protein.

^†^The best-matching viruses were determined by using the Basic Local Alignment Search Tool (BLAST) to find the highest identity between nucleotide query sequence and all publicly available DNA sequences in the website (https://www.gisaid.org/) of Global Initiative on Sharing All Influenza Data (GISAID). Only the virus with the closet isolation date to the virus for query was selected to be listed while more than one virus with the highest identity has been searched.

^‡^Accession numbers are listed in square brackets.

Table S4. Variations in hemagglutinin and neuraminidase* of 3 swine strains of influenza B virus compared with B/Brisbane/60/2008

| Virus strains | Amino acid position^†^ of HA | | | | |  | Amino acid position^†^ of NA | | | | |
| --- | --- | --- | --- | --- | --- | --- | --- | --- | --- | --- | --- |
|  | 124 | 146 | 197 | 424 | 525 |  | 172 | 295 | 340 | 358 | 369 |
| B/Brisbane/60/2008^‡^ | V | I | N | I | D |  | F | S | N | E | M |
| B/swine/Pingtung/58-15/2014 | A | V | D | -^§^ | E |  | - | R | D | K | I |
| B/swine/Tainan/77-5/2014 | A | V | D | M | E |  | L | R | D | K | I |
| B/swine/Taoyuan/78-1/2014 | A | V | D | M | E |  | - | R | D | K | I |

*Amino acid sequences of HA protein without signal peptide (15 amino acids) were compared. Accession numbers for HA and NA genes: B/Brisbane/60/2008 (FJ766840 and FJ766839), B/swine/Pingtung/58-15/2014 (MG002410 and MG002412), B/swine/Tainan/77-5/2014 (MG002418 and MG002420) and B/swine/Taoyuan/78-1/2014 (MG002426 and MG002428).

^†^The residues are numbered according to B/Brisbane/60/2008.

^‡^Vaccine strain used in 2009-12, Taiwan.^12^

^§^The en-dash (-) stands for identical amino acid to B/Brisbane/60/2008.

Reference:

1. Poon LL, Chan KH, Smith GJ, et al. Molecular detection of a novel human influenza (H1N1) of pandemic potential by conventional and real-time quantitative RT-PCR assays. *Clin Chem.* 2009;55(8):1555-1558.

2. Choi YK, Goyal SM, Kang SW, Farnham MW, Joo HS. Detection and subtyping of swine influenza H1N1, H1N2 and H3N2 viruses in clinical samples using two multiplex RT-PCR assays. *J Virol Methods.* 2002;102(1-2):53-59.

3. Choi YK, Goyal SM, Joo HS. Evaluation of a multiplex reverse transcription-polymerase chain reaction assay for subtyping hemagglutinin genes 1 and 3 of swine influenza type A virus in clinical samples. *J Vet Diagn Invest.* 2002;14(1):62-65.

4. Lee MS, Chang PC, Shien JH, Cheng MC, Shieh HK. Identification and subtyping of avian influenza viruses by reverse transcription-PCR. *J Virol Methods.* 2001;97(1-2):13-22.

5. Takao S, Shimazu Y, Fukuda S, Kuwayama M, Miyazaki K. Neuraminidase subtyping of human influenza a viruses by RT-PCR and its application to clinical isolates. *Jpn J Infect Dis.* 2002;55(6):204-205.

6. Tsai HP, Wang HC, Kiang D, et al. Increasing appearance of reassortant influenza B virus in Taiwan from 2002 to 2005. *J Clin Microbiol.* 2006;44(8):2705-2713.

7. Lee EY, Lee KH, Jung EJ, et al. Genotyping and screening of reassortant live-attenuated influenza B vaccine strain. *J Virol Methods.* 2010;165(2):133-138.

8. Hoffmann E, Mahmood K, Yang CF, Webster RG, Greenberg HB, Kemble G. Rescue of influenza B virus from eight plasmids. *Proc Natl Acad Sci U S A.* 2002;99(17):11411-11416.

9. Jumat MR, Sugrue RJ, Tan BH. Genetic characterisation of influenza B viruses detected in Singapore, 2004 to 2009. *BMC Res Notes.* 2014;7:863.

10. Tewawong N, Suwannakarn K, Prachayangprecha S, et al. Molecular epidemiology and phylogenetic analyses of influenza B virus in Thailand during 2010 to 2014. *PLoS One.* 2015;10(1):e0116302.

11. Byarugaba DK, Erima B, Millard M, et al. Genetic analysis of influenza B viruses isolated in Uganda during the 2009-2010 seasons. *Virol J.* 2013;10:11.

12. Kuo SM, Chen GW, Velu AB, et al. Circulating pattern and genomic characteristics of influenza B viruses in Taiwan from 2003 to 2014. *J Formos Med Assoc.* 2016;115(7):510-522.
